# SPECT/CT confirms metastatic pulmonary calcification on bone scintigraphy: Directing management in a dialysis patient

**DOI:** 10.1016/j.radcr.2026.07.117

**Published:** 2026-09-05

**Authors:** Xin Su, Weigang Li, Jin He, Haijun Wang

**Affiliations:** a Department of Nuclear Medicine, Gansu Provincial Hospital, Lanzhou 730000, China; b Department of Nuclear Medicine, Dingxi People's Hospital, Dingxi 743000, China; c Department of Critical Care Medicine, Gansu Provincial Hospital, Lanzhou 730000, China

**Keywords:** Bone scintigraphy, Metastatic calcification, Renal osteodystrophy, SPECT/CT, Clinical decision-making

## Abstract

We report a case of metastatic pulmonary calcification diagnosed by ^99m^Tc-MDP bone scintigraphy with SPECT/CT in a 52-year-old man with end-stage renal disease on hemodialysis. The patient presented with bone pain and mild cough, and had severe biochemical derangements of chronic kidney disease-mineral and bone disorder. This definitive objective evidence directly prompted immediate escalation of medical therapy. The case highlights the pivotal role of functional imaging in both diagnosing this condition and catalyzing aggressive clinical management.

## Introduction

Chronic kidney disease-mineral and bone disorder (CKD-MBD) is a common and serious complication in end-stage renal disease (ESRD) patients, characterized by systemic disturbances in mineral metabolism, leading to renal osteodystrophy and ectopic calcification. Metastatic calcification, particularly in the lungs, is a recognized manifestation of advanced CKD-MBD, which can be associated with significant morbidity [1]. The term “metastatic” refers to the metabolic origin of the calcification (dystrophic/calcium-phosphate deposition) rather than neoplastic malignant dissemination. While diagnosis traditionally relies on biochemical markers and computed tomography (CT), ^99m^Tc-methylene diphosphonate (MDP) bone scintigraphy, especially when combined with single-photon emission computed tomography/computed tomography (SPECT/CT), offers a unique functional and anatomical assessment, capable of detecting active calcific deposits before they become morphologically evident [2,3]. This case highlights the pivotal role of bone scintigraphy with SPECT/CT in diagnosing pulmonary metastatic calcification and its direct impact on guiding aggressive clinical management.

## Case report

A 52-year-old man with a 12-year history of maintenance hemodialysis for ESRD presented with progressive bone pain and a mild, nonproductive cough over 6 months. Laboratory evaluation revealed serum creatinine: 1018.80 μmol/L (normal range 57-97), serum calcium of 2.57 mmol/L (2.10-2.60), phosphate 2.14 mmol/L (0.85-1.51), alkaline phosphatase 320 U/L (40-130), OSTECO >300.00 ng/mL (14-46), and PTH 317 pg/mL (12-88). Estimated GFR was <15 mL/min/1.73 m². The patient had a 30-year history of hypertension, no diabetes mellitus, and no family history of relevant diseases. Collectively, laboratory abnormalities demonstrated significant hyperphosphatemia and markedly elevated parathyroid hormone. A ^99m^Tc-MDP whole-body bone scan was performed for further investigation.

**Fig. 1:**
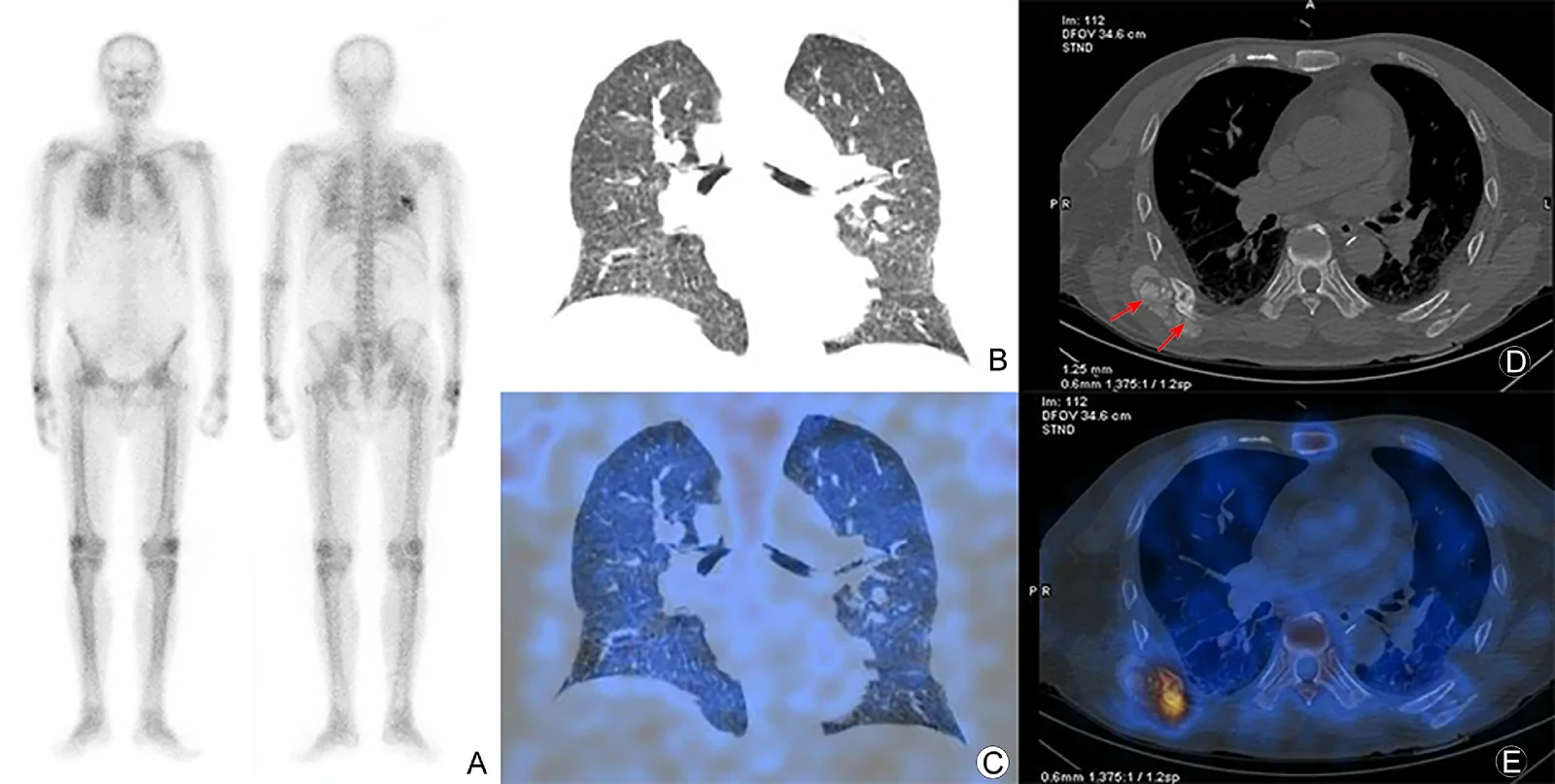
^99m^Tc-MDP imaging findings of systemic ectopic calcification. (A) Whole-body planar images (anterior and posterior) show a superscan pattern with diffuse bilateral pulmonary uptake. (B and C) Fused SPECT/CT of the chest localizes the uptake to lung parenchymal opacities, confirming metastatic pulmonary calcification. (D and E) Fused SPECT/CT of the right shoulder shows uptake corresponding to right eighth posterior rib fracture with adjacent soft-tissue calcification (arrow), diagnostic of myositis ossificans.

Whole-body planar images (Fig. 1A) showed a “superscan” pattern with diffusely increased skeletal tracer uptake. Notably, there was intense, homogeneous, and diffuse radiotracer accumulation throughout both lung fields, a finding atypical for normal bone scan. To characterize this extraskeletal uptake, thoracic SPECT/CT was performed. Fused SPECT/CT images (Fig. 1B and C) precisely localized the abnormal tracer uptake to the lung parenchyma, corresponding to subtle, diffuse ground-glass and consolidative opacities on the low-dose CT component, diagnostic of metastatic pulmonary calcification. An incidental finding of intense focal uptake adjacent to the right scapula was seen, which on corresponding CT (Fig. 1D and E) corresponded to a rib fracture with adjacent soft-tissue calcification, consistent with myositis ossificans—another manifestation of ectopic calcification.

Based on the definitive SPECT/CT findings confirming systemic, severe ectopic calcification, the patient’s mild respiratory symptoms were considered likely related to metastatic pulmonary involvement. The patient subsequently underwent the parathyroid ultrasound examination. The ultrasound revealed bilateral hypoechoic nodules suggestive of hyperplasia (left upper: 14 × 6 mm, left lower: 20 × 11 mm, right upper: 18 × 12 mm, right lower: 20 × 10 mm. Parathyroidectomy was recommended based on these findings and the severely elevated PTH level, but the patient declined referral for surgical intervention and was managed with escalation of phosphate binder therapy and initiation of a calcimimetic agent (etelcalcetide). At the 3-month follow-up, the patient reported partial relief of bone pain symptoms.

## Discussion

This case exemplifies a classic but clinically significant finding on bone scintigraphy in advanced CKD-MBD:diffuse bilateral pulmonary uptake of ^99m^Tc-MDP, a hallmark of metastatic pulmonary calcification. This condition results from calcium-phosphate complex deposition in alveolar septa due to an elevated product. 99mTc-MDP binds to these microcrystalline calcium deposits via chemisorption, enabling their visualization on bone scintigraphy. In addition to the classic findings of uniformly increased skeletal uptake with diminished renal activity, the superscan pattern in this case must be interpreted in the context of ESRD, where reduced urinary excretion of the tracer may contribute to a relatively higher background soft-tissue activity. Despite this, the pulmonary uptake remained distinctly abnormal and disproportionate to background [4]. The principal differential diagnoses for such a pattern include pulmonary edema, amyloidosis, or diffuse metastatic disease; however, the specific distribution, clinical context of ESRD with severe secondary hyperparathyroidism, and corroborative SPECT/CT findings make metastatic calcification the definitive diagnosis [5].

SPECT/CT provides critical value in this context by precisely localizing tracer uptake to the lung parenchyma and confirming its correspondence to CT-visible calcific densities [5]. As demonstrated in this case, objective imaging evidence of end-organ (lung) damage served as a powerful catalyst for clinical action [6]. While biochemical abnormalities (hyperphosphatemia, elevated PTH) typically trigger treatment, visualization of actual tissue calcification provided unambiguous justification for immediate and escalated therapy, including pharmacotherapy intensification and surgical referral, which might have been delayed if based on labs alone [7]. Although the patient did not undergo parathyroidectomy, pharmacotherapy still alleviated bone pain and provided clinical benefit. Early detection of subclinical MPC by functional imaging may carry prognostic significance, allowing therapeutic intervention before the development of irreversible pulmonary compromise, thereby reinforcing the argument for routine surveillance imaging in high-risk CKD-MBD patients [8]. The incidental discovery of myositis ossificans further underscores the systemic nature of ectopic calcification and the unique capability of whole-body bone scintigraphy to provide a comprehensive assessment of CKD-MBD burden. This case is unique in demonstrating concurrent myositis ossificans and MPC on bone scintigraphy, a rare combination. It also shows that even without parathyroidectomy, aggressive pharmacotherapy can alleviate symptoms—a clinically relevant lesson for nonsurgical candidates [9].

## Conclusion

^99m^Tc-MDP whole-body bone scintigraphy, especially with SPECT/CT fusion, is a highly sensitive and specific imaging tool for the detection of metastatic pulmonary calcification in patients with advanced CKD-MBD. It provides definitive diagnostic confirmation, aids in excluding other pathologies, and most significantly, offers objective evidence of disease severity that can directly motivate and accelerate intensive clinical management. This case reinforces the importance of recognizing this classic imaging pattern and leveraging functional imaging to improve patient care in nephrology.

## Patient consent

The patient provided written informed consent for the publication of this case report, including all accompanying images and clinical data. All information has been anonymized to protect patient privacy.
